# Benzimidazoles Downregulate Mdm2 and MdmX and Activate p53 in MdmX Overexpressing Tumor Cells

**DOI:** 10.3390/molecules24112152

**Published:** 2019-06-07

**Authors:** Zuzana Mrkvová, Stjepan Uldrijan, Antonio Pombinho, Petr Bartůněk, Iva Slaninová

**Affiliations:** 1 Department of Biology, Faculty of Medicine, Masaryk University, Kamenice 5, Building A6, 62500 Brno, Czech Republic; zuzana.mrkvova@seznam.cz (Z.M.); uldrijan@med.muni.cz (S.U.); 2 International Clinical Research Center, St. Anne’s University Hospital, 65691 Brno, Czech Republic; 3 CZ-OPENSCREEN: National Infrastructure for Chemical Biology, Institute of Molecular Genetics AS CR, v. v. i., 14220 Prague, Czech Republic; antonio.pombinho@ibmc.up.pt (A.P.); bartunek@img.cas.cz (P.B.)

**Keywords:** benzimidazoles, drug repurposing, Mdm2, MdmX, melanoma, p53

## Abstract

Tumor suppressor p53 is mutated in about 50% of cancers. Most malignant melanomas carry wild-type p53, but p53 activity is often inhibited due to overexpression of its negative regulators Mdm2 or MdmX. We performed high throughput screening of 2448 compounds on A375 cells carrying p53 activity luciferase reporter construct to reveal compounds that promote p53 activity in melanoma. Albendazole and fenbendazole, two approved and commonly used benzimidazole anthelmintics, stimulated p53 activity and were selected for further studies. The protein levels of p53 and p21 increased upon the treatment with albendazole and fenbendazole, indicating activation of the p53–p21 pathway, while the levels of Mdm2 and MdmX decreased in melanoma and breast cancer cells overexpressing these proteins. We also observed a reduction of cell viability and changes of cellular morphology corresponding to mitotic catastrophe, i.e., G2/M cell cycle arrest of large multinucleated cells with disrupted microtubules. In summary, we established a new tool for testing the impact of small molecule compounds on the activity of p53 and used it to identify the action of benzimidazoles in melanoma cells. The drugs promoted the stability and transcriptional activity of wild-type p53 via downregulation of its negative regulators Mdm2 and MdmX in cells overexpressing these proteins. The results indicate the potential for repurposing the benzimidazole anthelmintics for the treatment of cancers overexpressing p53 negative regulators.

## 1. Introduction

The critical role of the tumor suppressor p53 is illustrated by the fact that nearly 50% of human malignant neoplasms carry mutations in the p53 gene (*TP53*). Additionally, its function is abrogated by the overexpression of Mdm2 and MdmX in a significant proportion of tumors retaining wild-type p53 [1,2]. Mdm2 serves as an E3 ubiquitin ligase that targets p53 for proteasome-mediated degradation [3]. MdmX and Mdm2 can dimerize via their RING finger domains, and this interaction regulates cellular levels of p53 and MdmX, as well as Mdm2 [1,4]. Amplification or overexpression of Mdm2 and MdmX occur in a wide range of tumors, including malignant melanoma [5]. Notably, MdmX overexpression was detected in approximately 65% of melanomas in stages I–IV [6]. Given that mutations of p53 are rare while MdmX overexpression frequently occurs in melanoma, targeting MdmX in these tumors could be of particular clinical relevance.

Malignant melanoma is an aggressive form of skin cancer with approximately 133,000 newly diagnosed cases worldwide each year. It is challenging to treat melanoma in the metastatic stage. The more the disease develops, the lower the five-year survival rate becomes: Starting at 100% during isolated horizontal growth and finishing at 7–19% during the expansion of distant metastases [7]. Current therapeutic approaches include surgical resection, chemotherapy based on alkylating agent dacarbazine, photodynamic therapy, and immunotherapy using interferon α-2b, interleukin-2, and anti-PD-1 antibodies nivolumab and pembrolizumab. More recently, new therapeutic targets have been identified based on the genetic changes affecting the ERK MAPK signaling pathway in melanocytes. The personalized therapeutic approach is based on the genetic investigation and application of the targeted therapy, such as small molecule inhibitors or antibodies that affect the mutated proteins. The BRAF gene is mutated in almost 50% of all melanomas. Vemurafenib and dabrafenib, selective BRAF-mutant inhibitors, were approved by the FDA for the treatment of melanomas carrying the most common activating mutation BRAF^V600E^ [8]. Despite these advances in therapy, its efficiency is frequently decreased due to the development of drug resistance and new treatment strategies need to be found. One of the promising approaches to the development of effective cancer therapy is drug repositioning (drug repurposing), i.e., redirecting existing drugs to new indications [9]. When compared to the approval process of new drugs, the benefits of this approach comprise lower expenses and shorter processing time since the toxicology studies have already been done.

**Figure 1:**
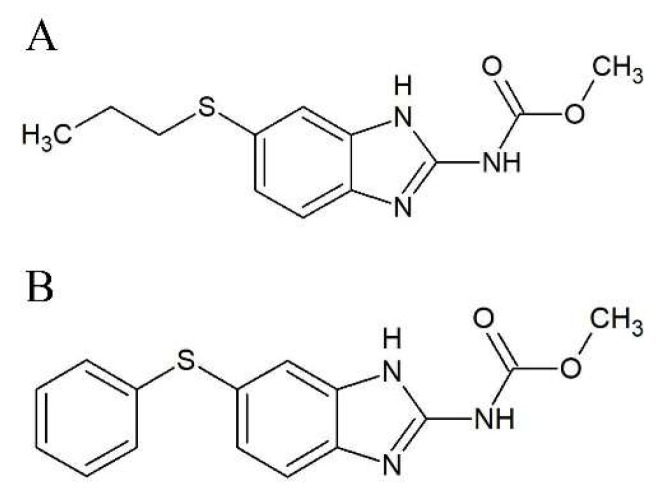
Chemical structures of (**A**) albendazole and (**B**) fenbendazole.

We performed high-throughput screening of more than 2000 pharmacologically relevant compounds in order to identify agents with the potential to increase the p53 transcriptional activity in human melanoma cells expressing high levels of MdmX. Based on the results, we selected two benzimidazole compounds, albendazole (ABZ, Figure 1A) and fenbendazole (FBZ, Figure 1B) for further investigations. Benzimidazoles are used as anthelmintics in veterinary and human medicine. The primary mechanism of their action is the disruption of the cellular microtubular system, the same mechanism by which two widely used groups of cytostatics, the Vinca alkaloids and taxanes, operate. Benzimidazoles bind to the colchicine-binding site in tubulin [10]. Since they bind to their own distinct binding sites, they can overcome the resistance to the cytostatics mentioned above [11]. ABZ could suppress the growth of paclitaxel-resistant leukemia (CEM/dEpoB300) and ovarian (1A9PTX22) tumor cells [12,13]. The response of paclitaxel-resistant cells to ABZ was even enhanced compared to the parental leukemia cells [12].

ABZ is the most studied benzimidazole derivative. It was developed in 1975 as an anthelmintic and was introduced to human medicine in 1982 [14]. The mechanism of ABZ action includes not only the microtubule inhibition but also the blockage of glucose uptake and generation of ROS, leading to low cellular levels of ATP [15] and DNA damage [16,17], respectively. Several authors published data supporting ABZ repositioning for cancer therapy. ABZ showed antiproliferative effects in cancer cell lines established from Ehrlich ascitic carcinoma [16], acute lymphoblastic leukemia [12], hepatocellular carcinoma [18], and colorectal carcinoma [19]. Regarding FBZ, Gao et al. observed the growth inhibition of lymphoma xenografted into SCID mice caused by a fenbendazole-containing diet combined with a high dose of vitamins [20].

In the light of the findings that malignant melanoma belongs to tumors retaining wild-type p53 but its function is limited by the overproduction of its negative regulators Mdm2 and MdmX, we searched for compounds that could restore p53 activity by targeting Mdm2 and MdmX. Here, we demonstrate that two clinically approved anthelmintics ABZ and FBZ increase p53 activity in cancer cells by downregulating Mdm2 and MdmX expression. In addition, we confirmed the antiproliferative activity of ABZ and FBZ, the disruption of microtubules, cell cycle arrest at the G2/M checkpoint, and changes in cellular morphology corresponding to the mitotic catastrophe.

## 2. Results

### 2.1. Effect on p53 Activity 

**Figure 2:**
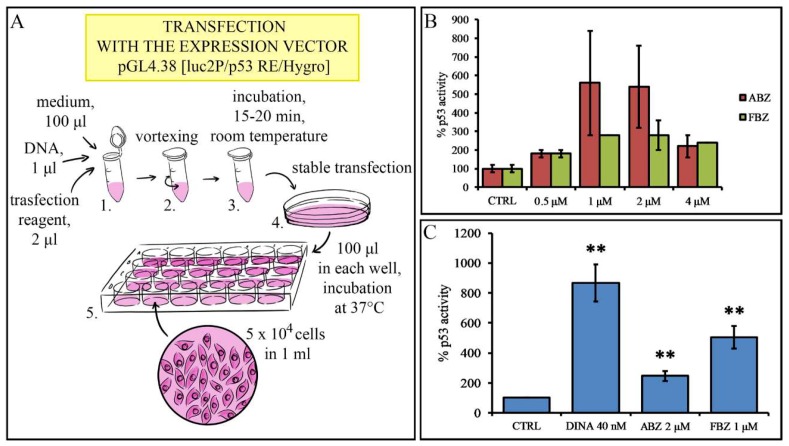
Luciferase assay of A375 melanoma cell line carrying a p53 activity luciferase reporter construct. (**A**) Scheme of HTS screening of 2448 compounds. (**B**) HTS revealed albendazole (ABZ) and fenbendazole (FBZ) as compounds that activate p53. (**C**) ABZ (2 μM) and FBZ (1 μM) significantly increased the activity of p53, dinaciclib (DINA) (40 nM) was used as a positive control. Solvent (DMSO) treated cells were used as a negative control (CTRL). The data are the means ± S.E.M. of three independent experiments; **p < 0.01.

In order to investigate the effect of small molecule drugs on p53 transcriptional activity in melanoma, we established A375 cell line carrying p53 activity luciferase reporter construct (A375-p53-Luc cells) and performed luciferase reporter assays (Figure 2A). In the initial screen of 2448 compounds, the stably transfected cells were treated with each of the tested compounds alone and in combination with the DNA damaging drug doxorubicin. The results were obtained by pilot screen, dose-response data, and data obtained by compounds that affected the p53 activity in A375-p53-Luc cells are presented in the supplement (Table S1, Table S2, and Figure S1). Based on the screening, we chose benzimidazole derivatives ABZ and FBZ for a more detailed study (Figure 2B). A375-p53-Luc cells were treated with ABZ (2 μM) and FBZ (1 μM) for 24 h. We and others have previously identified CDK inhibitors as potent non-genotoxic activators of p53 in melanoma cells [21,22]. For this reason, CDK inhibitor dinaciclib (DINA; 40 nM) was included as a positive control in our experiments. The results showed that DINA increased p53 activity to 865%, ABZ to 247%, and FBZ to 504% when compared to solvent (DMSO)-treated control cells (Figure 2C).

### 2.2. Mechanism of ABZ and FBZ Action

**Figure 3:**
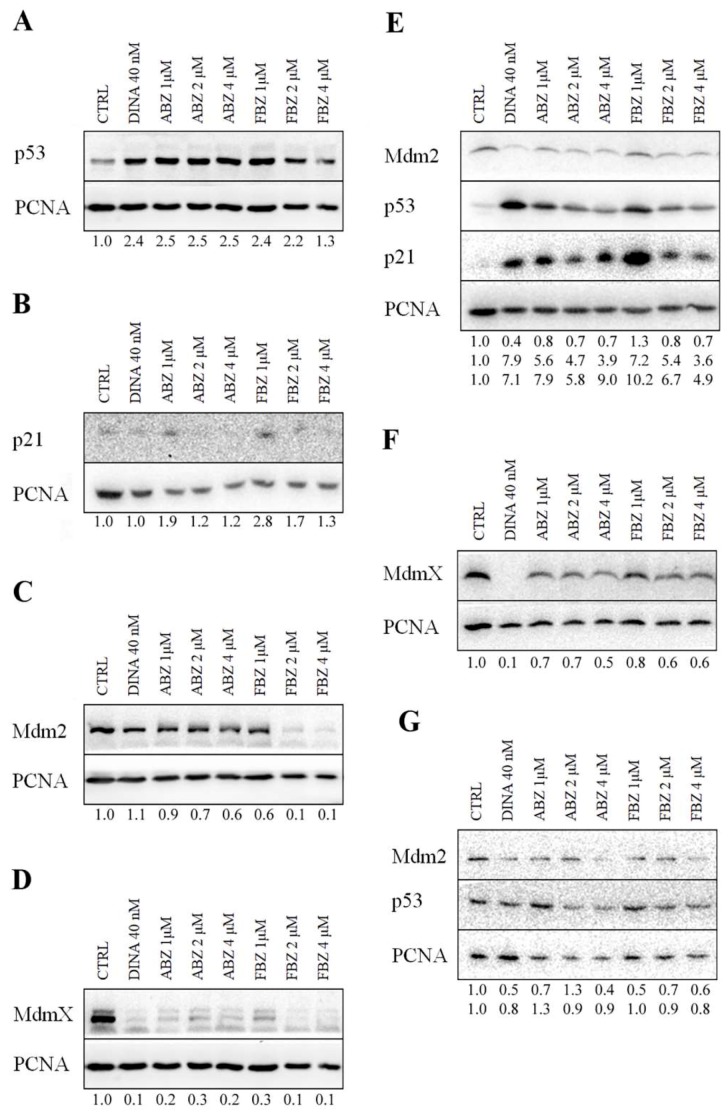
Effect of benzimidazoles on p53 and related proteins levels. DINA (40 nM) was used as a positive control. Solvent (DMSO)-treated cells were used as a negative control (CTRL). (**A**–**D**) WB analysis of A375 cells. (**A**) The A375 cells treated 24 h with ABZ (1, 2, and 4 μM), FBZ (1 and 2 μM), and DINA (40 nM) revealed p53 stabilization. (**B**) ABZ (1 μM) and FBZ (1 μM) increased the level of p21. (**C**) FBZ (2 μM and 4 μM) decreased the level of Mdm2, ABZ at all concentrations and FBZ (1 μM) had a weaker effect, and DINA did not affect p21. (**D**) The level of MdmX was decreased upon the treatment with DINA (40 nM), ABZ, and FBZ at concentrations 1, 2, and 4 μM. (**E**–**F**) Similar results were also obtained with MCF7 breast carcinoma cells. (**E**) p53 stabilization, an increase of p21 and decrease of Mdm2 levels was detected in DINA, ABZ (1, 2, and 4 μM) and FBZ (2 and 4 μM). (**F**) The decrease of MdmX levels was most pronounced in response to DINA, less after ABZ and FBZ (1, 2, and 4 μM) treatment. **(G**) In non-cancerous HFF cells, the response was milder, p53 was slightly stabilized upon ABZ (1 μM) treatment. The level of Mdm2 was decreased in ABZ (1 and 4 μM), and FBZ (1, 2, and 4 μM). MdmX levels were below the detection limit even in the control HFF cells. Total cell lysates were separated on 12.5% SDS gel. Proliferating cell nuclear antigen (PCNA) levels served as a loading control. Numeric values represent the ratio of band densities of the protein of interest normalized to the corresponding PCNA and the control normalized to the corresponding PCNA.

The benzimidazoles effects, particularly on p53, its major downstream target p21, and two main negative p53 regulators Mdm2 and MdmX were investigated by western blot analysis. Western blot analysis of A375 cells treated for 24 h with DINA (40 nM), ABZ (1, 2, and 4 μM) and FBZ (1 and 2 μM) showed approximately a 2.5-fold increase in p53 protein levels, while FBZ at the highest concentration (4 μM) had only a slight effect (1.3 fold) (Figure 3A). The level of p53 downstream effector p21 was the most significantly increased in samples treated with FBZ at concentration 1 μM (2.8 fold), while FBZ at concentration 2 μM (1.7 fold) and ABZ at concentration 1 μM (1.9 fold) had a weaker effect. This result indicated p53 activation, especially at lower concentrations of benzimidazoles (Figure 3B). Next, we studied the mechanism of p53 activation by examining the protein levels of the two main negative p53 regulators Mdm2 and MdmX. Significantly decreased levels of Mdm2 were observed only in samples treated with FBZ (2 and 4 μM, 0.1 fold). Interestingly, the effect on MdmX was much more pronounced, the levels of MdmX were significantly decreased upon the treatment with DINA and with both benzimidazoles (0.1–0.2 fold) (Figure 3C,D).

Similar to melanoma cells, the expression of Mdm2 and MdmX is often increased in breast carcinoma cells. Therefore, we investigated the effect of benzimidazoles on MCF7 cells overexpressing both proteins. In accordance with our previous results, WB analysis of MCF7 cells treated for 24 h also showed p53 stabilization most significant in response to DINA (7.8 fold) and FBZ at concentration 1 μM (7.2 fold), but also in cells treated with ABZ at concentrations 1, 2, and 4 μM (5.6–3.9 fold) and FBZ at concentrations 2 and 4 μM (5.4; 3.6 fold). The p53 stabilization was indirectly concentration-dependent, the higher concentrations of benzimidazoles caused weaker stabilization. The increase in p21 levels upon 24 h treatment with ABZ and FBZ at concentrations 1, 2, and 4 μM was more pronounced than in A375 cells, FBZ led to 10.2; 6.7; 4.9 fold increase, again with the highest effect at the lowest concentration, while ABZ had the highest effect at a concentration 4 μM (9 fold) and 1 μM (7.9 fold) and slightly weaker effect at 2 μM concentration (5.8 fold) (Figure 3E). Mdm2 and MdmX levels were slightly decreased (0.7–0.8 fold) by both benzimidazoles with the exception of FBZ at concentration 1 μM, showing a slight increase (1.3 fold) (Figure 3F). It is apparent that the effect of benzimidazoles on p53 and its signaling pathway is higher at lower drug concentrations, possibly indicating additional or off-target effects at higher concentrations.

HFF cells were used as non-cancerous controls. We detected only a slight p53 stabilization in HFF upon the treatment with ABZ at 1 μM concentration (1.3 fold). The levels of Mdm2 were decreased upon the treatment with our compounds at all concentrations (0.4–0.7 fold) except for ABZ at the concentration 2 μM, where we detected a slight increase (1.3 fold) (Figure 3G). We could not detect any MdmX protein, even in the untreated controls. These results collectively confirm the potential of benzimidazoles for the activation of the p53 pathway in tumors overexpressing Mdm2 and MdmX.

### 2.3. Effect on Microtubules, Cell Cycle, and DNA

**Figure 4:**
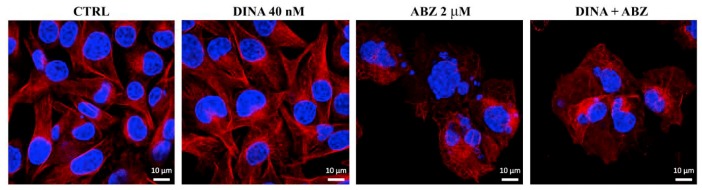
ABZ disrupts microtubules in A375 cells. Confocal laser scanning microscopy. Microtubules (**red**) stained by indirect immunofluorescence and nuclei (DAPI staining, **blue**) after 24 h treatment with ABZ (2 μM), DINA (40 nM), and a combination of both. The regular network of microtubules in control and DINA-treated cells. Thinned, disappearing, and irregular microtubules and multiple nuclei were apparent in cells treated with ABZ and the combination of ABZ with DINA. Bar = 10 μm.

Since benzimidazoles are well-known microtubule toxins, we studied the effects of ABZ on the microtubule cytoskeleton and the effect of benzimidazoles on the progression through the cell cycle. Control A375 cells and cells treated only with DINA (40 nM) showed a regular network of cytoplasmic microtubules. After a 24 h treatment with ABZ (2 μM), cells became enlarged, the microtubule network turned into an irregular tangle, and microtubules thinned out. Large cells were multinucleated, indicating defects in mitosis, i.e., the so-called mitotic catastrophe. The co-treatment with DINA had no impact on the ABZ effect on microtubules (Figure 4).

**Figure 5:**
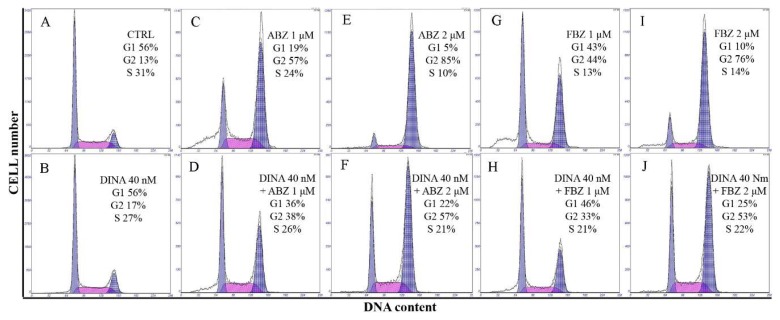
ABZ and FBZ arrest cell cycle at G2/M phase. Flow cytometry analysis of PI-stained cells. (**A**) Control, DMSO treated cells. (**B**) Cells treated with DINA. (**C**,**E**,**G**,**I**) ABZ and FBZ (1 and 2 μM) and their combinations with DINA (40 nM, **D**,**F**,**H**,**J**) significantly increased the proportion of A375 cells in G2/M phase. DINA alone had no significant effect on the cell cycle distribution.

DINA (40 nM) alone did not change the distribution of cell cycle phases in A375 cells. ABZ (1 and 2 μM) and FBZ (1 and 2 μM) and their combinations with DINA caused cell cycle arrest at the G2/M phase. The percentage of cells in the G2/M phase was lower in samples treated with combinations of DINA and benzimidazoles when compared to the treatments with benzimidazoles alone (Figure 5). This might be caused by the ability of DINA to interfere with the activity of multiple CDKs and stop the cell cycle in any phase.

**Figure 6:**
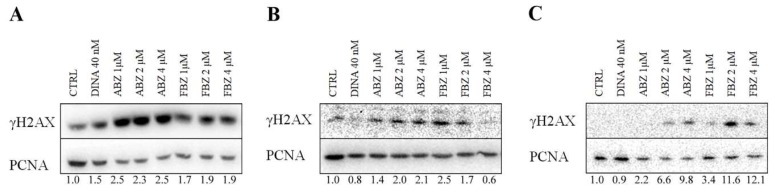
ABZ and FBZ caused DNA damage. WB analysis revealed increased levels of γH2AX in all (**A**) A375, (**B**) MCF7, and (**C**) HFF cells treated with ABZ and FBZ (1, 2, and 4 μM) for 24 h. Total cell lysates were separated on a 12.5% SDS gel. PCNA was used as a loading marker. Solvent (DMSO) treated cells were used as a negative control (CTRL). DINA (40 nM) that was used as a positive control affected H2AX phosphorylation only in A375 cells. Numeric values represent the ratio of band densities of the protein of interest normalized to the corresponding PCNA and the control normalized to the corresponding PCNA.

To determine if benzimidazoles caused DNA damage, we performed WB analysis of γH2AX, an indicator of double-strand DNA breaks. DINA (40 nM) affected H2AX phosphorylation only in A375 cells. After 24 h treatment with ABZ and FBZ at concentrations 1, 2, and 4 μM, we detected an increase in the level of γH2AX in A375 cell line in response to ABZ (2.3–2.5 fold increase), that was weaker in FBZ-treated cells (1.7–1.9 fold increase). The effect did not seem to be concentration-dependent. In MCF7 cells, ABZ showed a higher effect at higher concentrations (1.4–2.1 fold increase), while the FBZ effect decreased with increasing drug concentration (2.5–0.6 fold increase). The effect was most obvious in HFF cells, probably because of the complete absence of γH2AX signal in untreated controls. Both benzimidazoles increased the γH2AX levels in a concentration-dependent manner: ABZ induced a 2.2 to 9.8-fold increase and FBZ a 3.4 to 12.1 fold increase (Figure 6). This result is in accordance with our hypothesis that lower benzimidazoles concentrations could have a more specific effect on the p53 pathway compared to higher concentrations possibly causing off-target effects.

### 2.4. Effect on Cell Proliferation and Viability

**Figure 7:**
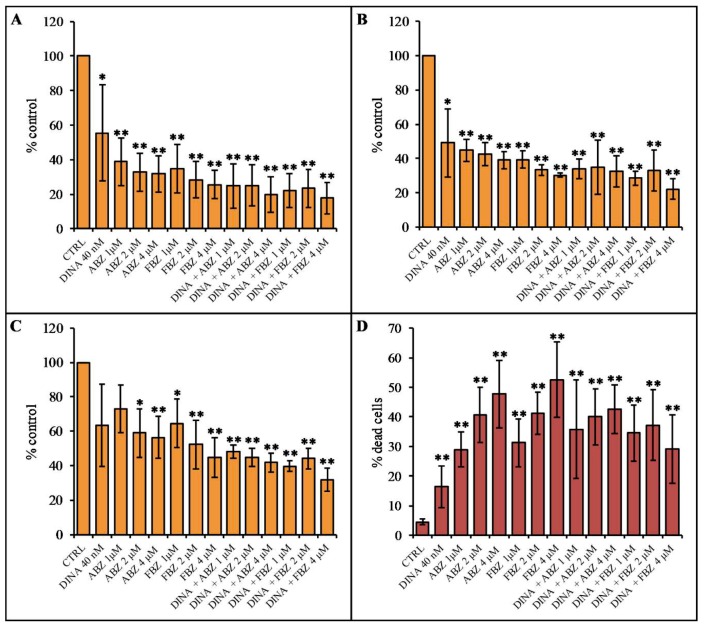
The effect of 48 h treatment with ABZ and FBZ on cell proliferation and viability. MTT assay of (**A**) A375 and (**B**) MCF and (**C**) HFF cells. (**D**) Flow cytometry analysis of PI-stained A375 cells. DINA (40 nM), ABZ (1, 2, and 4 μM), and FBZ (1, 2 and 4 μM) caused a significant decrease in the proliferation of A375 and MCF7 cells. A co-treatment with DINA and benzimidazoles did not have a significant impact on cell proliferation when compared to the treatments with individual compounds. The data are the means ± S.E.M. of three independent experiments; * *p* < 0.05; ** *p* < 0.01.

The antiproliferative activity of benzimidazoles was studied using MTT colorimetric assay and PI-exclusion assay. MTT assay detects cellular metabolic activity that is directly proportional to the number of living cells. The results showed a significant decrease in proliferation of A375 (Figure 7A) and MCF7 (Figure 7B) cell cultures treated for 48 h with ABZ and FBZ at 1, 2, and 4 μM concentrations. DINA decreased the proliferation to a lesser extent than the benzimidazole compounds. The effect was concentration-dependent in A375 cells, while in MCF, all concentrations of benzimidazoles had a similar effect. A co-treatment with DINA and benzimidazoles did not have a stronger impact on cell proliferation when compared to the treatments with individual compounds (Figure 7A,B). In non-cancerous HFF cells, benzimidazoles, DINA, and their combinations decreased cell proliferation to a lesser extent than in A375 and MCF7 cells (Figure 7C). This difference in the response of cancer and non-cancer cells was statistically significant upon treatment with 1 μM ABZ and the combinations of ABZ (1 and 4 μM) and DINA (40 nM) in the case of A375 cells and upon treatment with the combinations of ABZ 1 μM and DINA and FBZ 1 μM and DINA in the case of MCF7 cells. The PI-exclusion assay showed that 48 h treatments with DINA (40 nM), ABZ, and FBZ at concentrations 1, 2, and 4 μM significantly increased the proportion of dead A375 cells. As with the MTT experiments, benzimidazoles did not significantly potentiate the effect of DINA (Figure 7D).

The comparison of the results of MTT metabolic activity assay and PI-exclusion assay indicated that benzimidazoles might act in a two-step manner in A375 cells. Initially, they could stop cell proliferation, and subsequently, they could induce cell death. This finding corresponds with results showing the cell cycle arrest at G2/M mentioned above. 

## 3. Discussion

As the therapeutic options for metastatic melanoma are relatively limited, mostly due to the early metastases and intrinsic or acquired drug resistance, research into new possible treatment is of critical importance. Malignant melanoma is a tumor carrying predominantly a wild-type form of the tumor suppressor p53. However, its function is often restricted owing to the overexpression of its negative regulators Mdm2 and MdmX [1]. Targeting the activity of these regulators could restore p53 function and improve the efficiency of therapy.

Thus, we performed a high-throughput screening of more than two thousand agents to search for compounds with the ability to increase the p53 activity in malignant melanoma cells overexpressing MdmX. Among others, the screening revealed the promising activity of ABZ and FBZ, two approved and commonly used anthelmintic drugs [14]. The fact that they are also well-known microtubule toxins supported our decision to choose these benzimidazoles for further tests. 

There has been increasing evidence that benzimidazoles are attractive compounds for cancer therapy. Antiproliferative activity of ABZ and other benzimidazoles was described in a broad spectrum of cancer cell lines such as hepatocellular [18], colorectal [19], human HPV-negative HNSCC [23], as well as in melanoma cell lines. Doudican et al. demonstrated that mebendazole selectively induces Bcl2-mediated apoptosis in melanoma cells but not in melanocytes [24]. Čáňová et al. described the inhibitory effects of flubendazole on several melanoma cell lines [25]. In vivo experiments performed on nude mice proved the antitumor activity of ABZ [26]. 

The mechanism of benzimidazoles action in cancer cells has not been fully elucidated. As mentioned above, similar to widely used cytostatics such as Vinca alkaloids and taxanes, they are microtubular toxins and cause cell cycle arrest at the G2/M checkpoint. Additionally, other mechanisms of benzimidazoles action have already been described. These include the alteration of cell metabolism, ROS production, and DNA damage, all of which can lead to the destruction of cancer cells [16,17,27]. Pourgholami et al. demonstrated a reduction of VEGF and HIF-1α activity and inhibition of angiogenesis upon ABZ treatment [28,29]. FBZ caused mitochondrial translocation of p53 and inhibited glucose uptake as well as expression of GLUT transporters and hexokinase (HK II), a key glycolytic enzyme in cancer cell metabolism [30]. 

This work deals with the effect of ABZ and FBZ on the p53 pathway activity. There are several possible ways of p53 activation upon benzimidazole treatment. p53 as a guardian of the genome is activated in response to DNA damage. Benzimidazoles can cause DNA damage either indirectly by ROS production or directly by interaction with DNA [17] and the DNA helicase [31]. Castro et al. observed a potent antiproliferative effect of ABZ in MCF7 cells due to oxidative stress and the induction of DNA damage [16]. These authors also demonstrated that ABZ (20 mg/kg) inhibited tumor growth, increased cellular levels of p53 and the pro-apoptotic Bax protein, and decreased levels of the anti-apoptotic Bcl-xL in Ehrlich ascites carcinoma-bearing mice. Benzimidazoles may also affect p53 because of their ability to interact with microtubules. It was shown that p53 is transported into the nucleus by a microtubule-dependent dynein–dynactin motor complex after DNA damage and disruption of the microtubules network by microtubule toxins such as paclitaxel and vincristine stops this transport and inhibits activation of downstream targets of p53 [32,33]. Interestingly, low doses of microtubule toxins that do not cause microtubules disruption suppress the dynamics of microtubules, enhance nuclear accumulation and stabilization of p53, and increase the expression of Mdm2. In contrast, high doses disrupting the microtubule network stabilize p53 in the cytoplasm and decrease Mdm2 levels [33]. As benzimidazoles at doses used in our experiments disrupt microtubules network, the above-mentioned mechanism could be involved in the observed decrease of Mdm2, and probably also MdmX levels, and in the stabilization of p53.

Patel et al. showed that ABZ induces DNA damage in melanoma and small-cell lung cancer cells and sensitizes them to radiation [27]. In agreement with these results, we detected γH2AX upon treatment with ABZ and FBZ, clearly indicating the presence of DNA damage. Of note, benzimidazoles also sensitized V-79 cells to the cytotoxic effects of ionizing radiation in hypoxic conditions [34]. Contrary to previous findings, Duan et al. observed no effect of FBZ at antiparasitic doses on radiotherapy outcomes when tested on breast cancer inoculated mice [35]. However, the results of in vitro experiments on EMT6 cells with higher FBZ doses showed an antiproliferative effect. 

Using the luciferase reporter assay, we demonstrated that ABZ and FBZ increase the activity of p53. WB analysis proved increased levels of p53 and p21, proteins indicating the activation of the p53-p21 pathway. The elevated level of p53 could be caused by the activation of its expression, inhibition of p53 negative regulators or inhibition of its degradation mechanism. In reference to the latter mechanism, Dogra et al. observed increased p53 activity and its accumulation in nuclei upon FBZ treatment in H460 and A549 lung cancer cells [36]. This was not accompanied by a corresponding increase in the level of its mRNA. Recent findings of Dogra et al. show the importance of p53 in the response of cancer cells to benzimidazoles [30]. They demonstrated that tumor cell lines carrying wild-type p53 were highly sensitive to FBZ treatment when compared to p53 mutant or null cells. These findings are in accordance with our data suggesting a significant effect of benzimidazoles on the p53 pathway activity in cancer cells.

Here, we proved the antiproliferative activity of two benzimidazoles ABZ and FBZ in melanoma and breast cancer cell lines overexpressing MdmX. The novelty of the present study resides in the demonstration of ABZ and FBZ ability to downregulate the cellular levels of essential negative p53 regulators Mdm2 and especially MdmX in cancer cells. In accordance with the previously reported antimicrotubular activity of benzimidazoles, we observed changes in cellular morphology corresponding to mitotic catastrophe, i.e., G2/M cell cycle arrest of large multinucleated cells with disrupted microtubules. We detected γH2AX indicating DNA damage as well. Collectively, these disturbances of cellular physiology could lead to the induction of apoptosis in cancer cells. Indeed, apoptosis upon benzimidazoles treatment was described by several authors [19,25,27,35]. 

## 4. Materials and Methods 

### 4.1. Drugs and Reagents

Albendazole (methylN-(6-propylsulfanyl-1H-benzimidazol-2-yl) carbamate, Figure 1A), fenbendazole (methyl5-(phenylthio)-2-benzimidazolecarbamate, Figure 1B), Dinaciclib (DINA), MTT, Coomassie Brilliant Blue G-250, propidium iodide, trypsin, and DAPI were purchased from Sigma-Aldrich (Prague, Czech Republic), TurboFect and Luciferase Assay Reagent and Firefly Luciferase Glow Assay Kit and Bradford Reagent from Bio-Rad (Prague, Czech Republic). ECL chemiluminescence reagent was purchased from ThermoFisher Scientific (Waltham, MA, USA).

### 4.2. Cells

A375 (human malignant melanoma) and MCF7 (human breast adenocarcinoma) cell lines were purchased from ECACC, Salisbury, UK. HFF-1 cells (human foreskin fibroblasts) were obtained from ATCC. A375 and MCF7 cells were cultivated in RPMI 1640 and HFF cells in DMEM medium (HyClone Laboratories, Inc., Logan, UT, USA). Growth media were supplemented with 2 mM L-glutamine (PAA Laboratories, Pasching, Austria), 10% fetal calf serum, 100 IU/ml penicillin, and 100 µg/ml streptomycin (HyClone Laboratories, Inc., Logan, UT, USA). Cells were incubated at 37 °C in a high-humidity atmosphere with 5% CO_2_ and were passaged three times a week. 

### 4.3. Primary Screening 

A375 cells were stably transfected with the expression vector pGL4.38 [luc2P/p53 RE/Hygro] using TurboFect, as was described previously [37]. A375-p53-Luc cells were plated in white 384-well plates (Corning) at a density of 2,500 cells/25 μl/well using a Multidrop Combi dispenser (Thermo Scientific) and cultivated overnight. Library compounds were then added using pintool (V&P Scientific) coupled to a JANUS Automated Workstation (PerkinElmer) to a final concentration of 1 μM. The compound library included the Library of Pharmacologically Active Compounds (LOPAC1280, Sigma-Aldrich, Prague, Czech Republic), the Prestwick Chemical Library (Illkirch, France), and the NIH Clinical Trial Collection (NIH, Bethesda, Maryland, USA). A total of 2448 unique compounds were used in the course of HTS screening. Drugs were tested alone and in combination with a DNA damaging drug doxorubicin. The cells were cultivated for 24 h, luciferase production was determined by the One-GLO assay (Promega), and luminescence acquired by an EnVision microplate reader (PerkinElmer).

### 4.4. Luciferase Assay and Bradford Assay

In subsequent experiments, A375-p53-Luc cells grown in 24-well plates were treated with the tested drugs for 24 h and were lysed in 150 μl of the luciferase assay lysis buffer. Luminescence was measured on TriStar^2^ Multimode Reader LB 942 (Berthold Technologies, Wildbad, Germany) after adding 25 μl of Luciferase Assay Reagent to 15 μL of each lysate sample in 96-well plate using Pierce Firefly Luciferase Glow Assay Kit according to the manufacturer’s instructions. To determine sample protein concentration, 200 μl Bradford Reagent (acidified solution of Coomassie Brilliant Blue G-250) was added to 10 μl of lysate samples and colorimetric analysis was performed on DTX 880 Multimode detector (Beckman Coulter Inc. Prague, Czech Republic). The activity of p53 was calculated as the ratio of luminescence to protein concentration.

### 4.5. SDS-PAGE and Western Blot Analysis 

Cells were seeded at the concentration of 5 × 10^4^ cells/ml in 12-well plates and treated with the tested drugs for 24 h, collected, washed with PBS, and lysed in 2x Laemmli buffer. SDS-PAGE and semi-dry electrotransfer were performed and membranes were blocked for 1 hour in 5% nonfat dry milk in TBS and incubated with primary antibodies at 4 °C overnight. As primary antibodies, we used anti-MdmX (8c6) and anti-Mdm2 (Ab-1, IF2) mouse monoclonal antibodies (04-1555; OP46, both Merck-Millipore, Prague, Czech Republic), anti-H2A.X Phospho (Ser139) mouse mAb (clone 2f3, BioLegend, Koblenz, Germany), anti-PCNA (PC-10), anti-p21 (118) and anti-p53 (DO-1) mouse mAb (kindly provided by Bořivoj Vojtěšek, Masaryk Memorial Cancer Institute, Brno, Czech Republic). Instead of a secondary antibody, Mouse m-IgGκ BP-HRP (sc-516102 Santa Cruz Biotechnology, Heidelberg, Germany) was applied for one hour. The signal was developed with an ECL chemiluminescence reagent and detected using G:BOX (Syngene, Cambridge, United Kingdom). 

### 4.6. MTT Assay

MTT assays were performed on 96-well plates (Nunc A/S, Roskilde, Denmark) as described previously [38]. Metabolic activity of cells treated with ABZ and FBZ (1-4 μM) and DINA (40 nM) was detected after 48 h of incubation. Each concentration of each compound was examined in four replicate wells. The experiments were independently repeated three times.

### 4.7. Propidium Iodide Exclusion Assay 

The cell viability assay was based on the exclusion of propidium iodide (PI) by the intact viable cells. Cells were plated in 12-well plates at 5 × 10^4^ cells/ml under standard conditions. Next day, cells were treated with ABZ and FBZ (1, 2, and 4 μM) and DINA (40 nM) for 48 h and collected by trypsinization. Immediately after PI addition, the percentage of dead (PI-positive) cells was determined using a Cytomics FC 500 flow cytometry system (Beckman Coulter, Inc., Prague, Czech Republic) on channel FL3 (emission at 620 nm). A total of 10,000 cells were analyzed for each sample. 

### 4.8. Immunofluorescence and DAPI Staining

A375 cells, grown 24 h on coverslips in the presence of ABZ (2 μM) and DINA (40 nM), were fixed with 3% paraformaldehyde in PBS for 20 min at room temperature, permeabilized with 0.2% Triton X-100 in PBS for 5 min and preincubated for 20 min in 0.5% BSA in PBS. Cells were stained with primary anti-β-tubulin (TU-01, Exbio, Prague, Czech Republic) antibody overnight. Alexa Fluor 594 Donkey Anti-Mouse IgG (A 21203, ThermoFisher Scientific, Waltham, MA, USA) was used as the secondary antibody. DAPI at a final concentration of 1 μg/ml was added to the solution of the secondary antibody for visualization of cell nuclei. Slides were observed using a confocal microscope (LSM 700, Carl Zeiss GmbH, Oberkochen, Germany).

### 4.9. Cell Cycle Analysis

Cells were seeded in 12-well plates as described above and treated with benzimidazoles (1 and 2 μM) and DINA (40 nM) for 24 h. Both detached and attached cells were harvested into ice-cold PBS by trypsinization, fixed in 70% ethanol for 30 min on ice, processed as described in [39] and analyzed using Cytomics FC 500 flow cytometer in the FL3 channel (emission wavelength 620 nm). Data from each sample were further analyzed for cell cycle phases using software Multicycle AV for Windows (Phoenix Flow system, San Diego, CA, USA). 

### 4.10. Statistics

Statistical analysis was carried out using the statistical program STATISTICA 12. Results were analyzed with Student’s t-test and the threshold for statistical significance was set to *p* < 0.01 and *p* < 0.05.

## 5. Conclusions

In this study, we established a new tool for testing of pharmacologically relevant compounds for their ability to enhance the activity of p53 in cancer cells. Our findings have several important implications. Benzimidazole derivatives ABZ and FBZ stimulate the activity of p53 in malignant melanoma and breast cancer cells overexpressing MdmX. Our data provide the first evidence that this enhancement occurs, at least in part, through MdmX and Mdm2 downregulation. This points out the significance of MdmX in p53 regulation in tumors that overexpress this essential negative p53 regulator, such as the majority of malignant melanomas. Both ABZ and FBZ induce a disruption of microtubules, DNA damage, cell cycle arrest in G2/M, and morphological features of mitotic catastrophe. These findings could lead to new perspectives for the repurposing of these anthelmintics, which are already approved for human treatment, for example, for therapy of the tumors overexpressing the negative p53 regulators Mdm2 and MdmX, including malignant melanoma.

## Supplementary Materials

The following are available online, Figure S1: Data obtained by compounds that affected the p53 activity in A375-p53-Luc cells, Table S1: Data obtained by pilot screen of A375-p53-Luc cells, Table S2: Data obtained by pilot screen of A375-p53-Luc cells.

## References

[1] Karni-Schmidt O., Lokshin M., Prives C. (2016). The roles of MDM2 and MDMX in cancer. Annu. Rev. Pathol..

[2] Toledo F., Wahl G.M. (2007). Review: MDM2 and MDM4: p53 regulators as targets in anticancer therapy. Int. J. Biochem. Cell Biol..

[3] Honda R., Tanaka H., Yasuda H. (1997). Oncoprotein MDM2 is a ubiquitin ligase E3 for tumor suppressor p53. Febs Lett..

[4] Shadfan M., Lopez-Pajares V., Yuan Z.-M. (2012). MDM2 and MDMX: Alone and together in regulation of p53. Transl. Cancer Res..

[5] Wade M., Li Y.-C., Wahl G.M. (2013). MDM2, MDMX and p53 in oncogenesis and cancer therapy. Nat Rev Cancer.

[6] Gembarska A., Luciani F., Fedele C., Russell E.A., Dewaele M., Villar S., Zwolinska A., Haupt S., de Lange J., Yip D. (2012). MDM4 is a key therapeutic target in cutaneous melanoma. Nat. Med..

[7] Stewart B.W., Wild C.P., Word Health Organization (2014). World Cancer Report 2014.

[8] Domingues B., Lopes J., Soares P., Populo H. (2018). Melanoma treatment in review. Immunotargets Ther..

[9] Telleria C.M. (2012). Drug repurposing for cancer therapy. J. Cancer Sci. Ther..

[10] Russell G.J., Lacey E. (1995). Inhibition of [3H] mebendazole binding to tubulin by structurally diverse microtubule inhibitors which interact at the colchicine binding site. Biochem. Mol. Biol. Int..

[11] Nogales E. (2000). Structural insights into microtubule function. Annu. Rev. Biochem..

[12] Khalilzadeh A., Wangoo K.T., Morris D.L., Pourgholami M.H. (2007). Epothilone-paclitaxel resistant leukemic cells CEM/dEpoB300 are sensitive to albendazole: Involvement of apoptotic pathways. Biochem. Pharmacol..

[13] Chu S.W.L., Badar S., Morris D.L., Pourgholami M.H. (2009). Potent inhibition of tubulin polymerisation and proliferation of paclitaxel-resistant 1A9PTX22 human ovarian cancer cells by albendazole. Anticancer Res..

[14] Upcroft P., Upcroft J. (2001). Drug targets and mechanisms of resistance in the anaerobic protozoa. Clin. Microbiol. Rev..

[15] Martin R.J. (1997). Modes of action of anthelmintic drugs. Vet. J..

[16] Castro L.S.E.P.W., Kviecinski M.R., Ourique F., Parisotto E.B., Grinevicius V.M.A.S., Correia J.F.G., Wilhelm Filho D., Pedrosa R.C. (2016). Albendazole as a promising molecule for tumor control. Redox Biol..

[17] Seaton A., Higgins C., Mann J., Baron A., Bailly C., Neidle S., van den Berg H. (2003). Mechanistic and anti-proliferative studies of two novel, biologically active bis-benzimidazoles. Eur. J. Cancer.

[18] Pourgholami M.H., Woon L., Almajd R., Akhter J., Bowery P., Morris D.L. (2001). In vitro and in vivo suppression of growth of hepatocellular carcinoma cells by albendazole. Cancer Lett..

[19] Králová V., Hanušová V., Rudolf E., Čáňová K., Skálová L. (2016). Flubendazole induces mitotic catastrophe and senescence in colon cancer cells in vitro. J. Pharm. Pharmacol..

[20] Gao P., Dang C.V., Watson J. (2008). Unexpected antitumorigenic effect of fenbendazole when combined with supplementary vitamins. J. Am. Assoc. Lab. Anim. Sci..

[21] Kotala V., Uldrijan S., Horky M., Trbusek M., Strnad M., Vojtesek B. (2001). Potent induction of wild-type p53-dependent transcription in tumour cells by a synthetic inhibitor of cyclin-dependent kinases. Cell. Mol. Life Sci..

[22] Desai B.M., Villanueva J., Nguyen T.-T.K., Lioni M., Xiao M., Kong J., Krepler C., Vultur A., Flaherty K.T., Nathanson K.L. (2013). The anti-melanoma activity of dinaciclib, a cyclin-dependent kinase inhibitor, is dependent on p53 signaling. PLoS ONE.

[23] Ghasemi F., Black M., Pinto N., Ruicci K.M., Yoo J., Fung K., MacNeil D., Mymryk J.S., Barrett J.W., Nichols A.C. (2017). Repurposing albendazole: New potential as a chemotherapeutic agent with preferential activity against HPV-negative head and neck squamous cell cancer. Oncotarget.

[24] Doudican N., Rodriguez A., Osman I., Orlow S.J. (2008). Mebendazole induces apoptosis via Bcl-2 inactivation in chemoresistant melanoma cells. Mol. Cancer Res..

[25] Čáňová K., Rudolf E., Rozkydalová L., Vokurková D. (2018). Flubendazole induces mitotic catastrophe and apoptosis in melanoma cells. Toxicol. in Vitro.

[26] Hanušová V., Králová V., Skálová L., Matoušková P. (2015). Potential anti-cancer drugs commonly used for other indications. Curr. Cancer Drug Targets.

[27] Patel K., Doudican N.A., Schiff P.B., Orlow S.J. (2011). Albendazole sensitizes cancer cells to ionizing radiation. Radiat Oncol.

[28] Pourgholami M.H., Cai Z.Y., Badar S., Wangoo K., Poruchynsky M.S., Morris D.L. (2010). Potent inhibition of tumoral hypoxia-inducible factor 1alpha by albendazole. BMC Cancer.

[29] Pourgholami M.H., Khachigian L.M., Fahmy R.G., Badar S., Wang L., Chu S.W.L., Morris D.L. (2010). Albendazole inhibits endothelial cell migration, tube formation, vasopermeability, VEGF receptor-2 expression and suppresses retinal neovascularization in ROP model of angiogenesis. Biochem. Biophys. Res. Commun..

[30] Dogra N., Kumar A., Mukhopadhyay T. (2018). Fenbendazole acts as a moderate microtubule destabilizing agent and causes cancer cell death by modulating multiple cellular pathways. Sci. Rep..

[31] Soderlind K., Gorodetsky B., Singh A., Bachur N., Miller G., Lown J. (1999). Bis-benzimidazole anticancer agents: Targeting human tumour helicases. Anti-Cancer Drug Des..

[32] Giannakakou P., Sackett D.L., Ward Y., Webster K.R., Blagosklonny M.V., Fojo T. (2000). p53 is associated with cellular microtubules and is transported to the nucleus by dynein. Nat. Cell Biol..

[33] Giannakakou P., Nakano M., Nicolaou K.C., O’Brate A., Yu J., Blagosklonny M.V., Greber U.F., Fojo T. (2002). Enhanced microtubule-dependent trafficking and p53 nuclear accumulation by suppression of microtubule dynamics. Proc. Natl. Acad. Sci. USA.

[34] Gupta R.P., Larroquette C.A., Agrawal K.C. (1982). Potential radiosensitizing agents. 5. 2-substituted benzimidazole derivatives. J. Med. Chem..

[35] Duan Y.-T., Wang Z.-C., Sang Y.-L., Tao X.-X., Zhu H.-L. (2013). Exploration of structure-based on imidazole core as antibacterial agents. Curr. Top Med. Chem..

[36] Dogra N., Mukhopadhyay T. (2012). Impairment of the ubiquitin-proteasome pathway by methyl N-(6-phenylsulfanyl-1H-benzimidazol-2-yl)carbamate leads to a potent cytotoxic effect in tumor cells: A novel antiproliferative agent with a potential therapeutic implication. J. Biol. Chem..

[37] Valianatos G., Valcikova B., Growkova K., Verlande A., Mlcochova J., Radova L., Stetkova M., Vyhnakova M., Slaby O., Uldrijan S. (2017). A small molecule drug promoting miRNA processing induces alternative splicing of MdmX transcript and rescues p53 activity in human cancer cells overexpressing MdmX protein. PLoS ONE.

[38] Hammerová J., Uldrijan S., Táborská E., Slaninová I. (2011). Benzo[c]phenanthridine alkaloids exhibit strong anti-proliferative activity in malignant melanoma cells regardless of their p53 status. J. Dermatol. Sci..

[39] Slaninová I., Březinová L., Koubíková L., Slanina J. (2009). Dibenzocyclooctadiene lignans overcome drug resistance in lung cancer cells—Study of structure–activity relationship. Toxicology in Vitro.

