# Supplementary material for: Benzimidazoles Downregulate Mdm2 and MdmX and Activate p53 in MdmX Overexpressing Tumor Cells
Source: Molecules. 2019 Jun 7;24(11):2152. doi: 10.3390/molecules24112152 (PMC6600429; doi:10.3390/molecules24112152)

p53-Luc Agonists

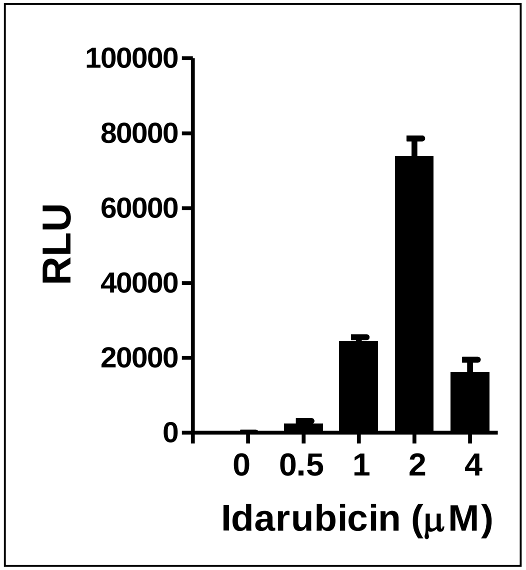

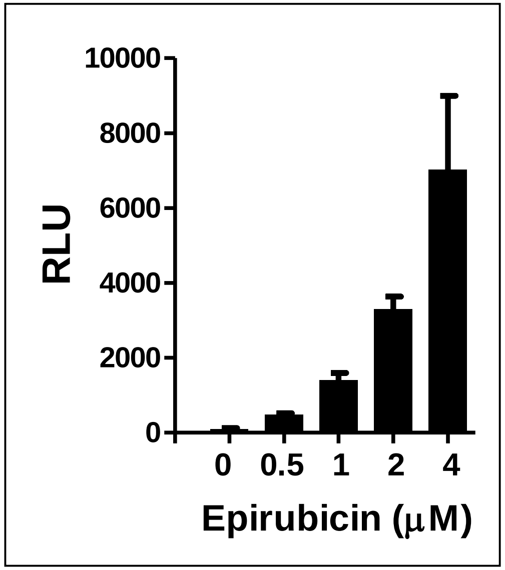

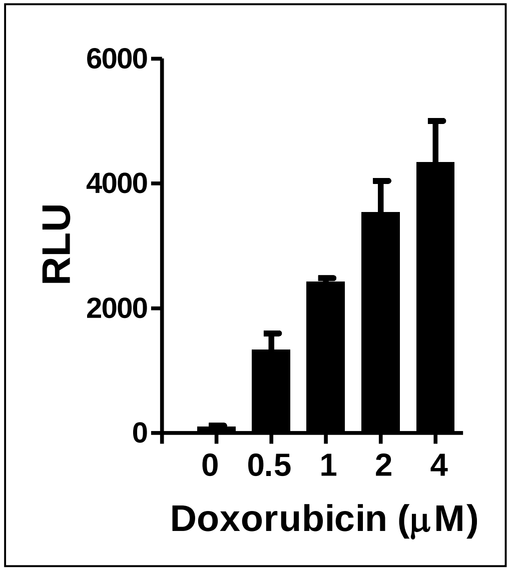

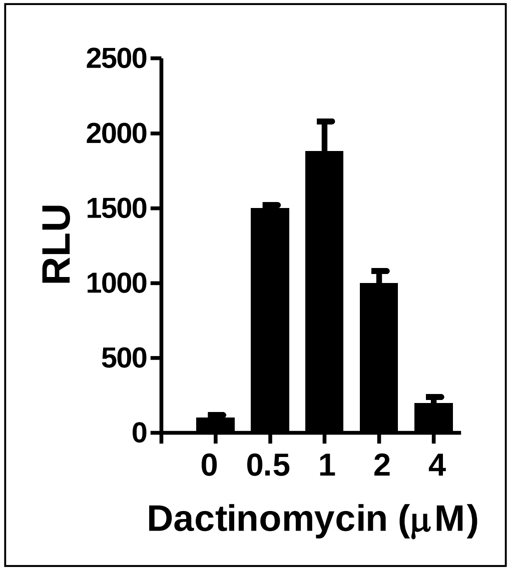

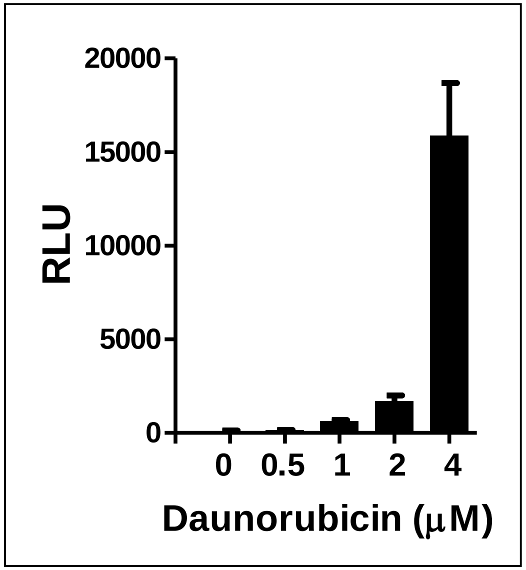

p53-Luc Agonists

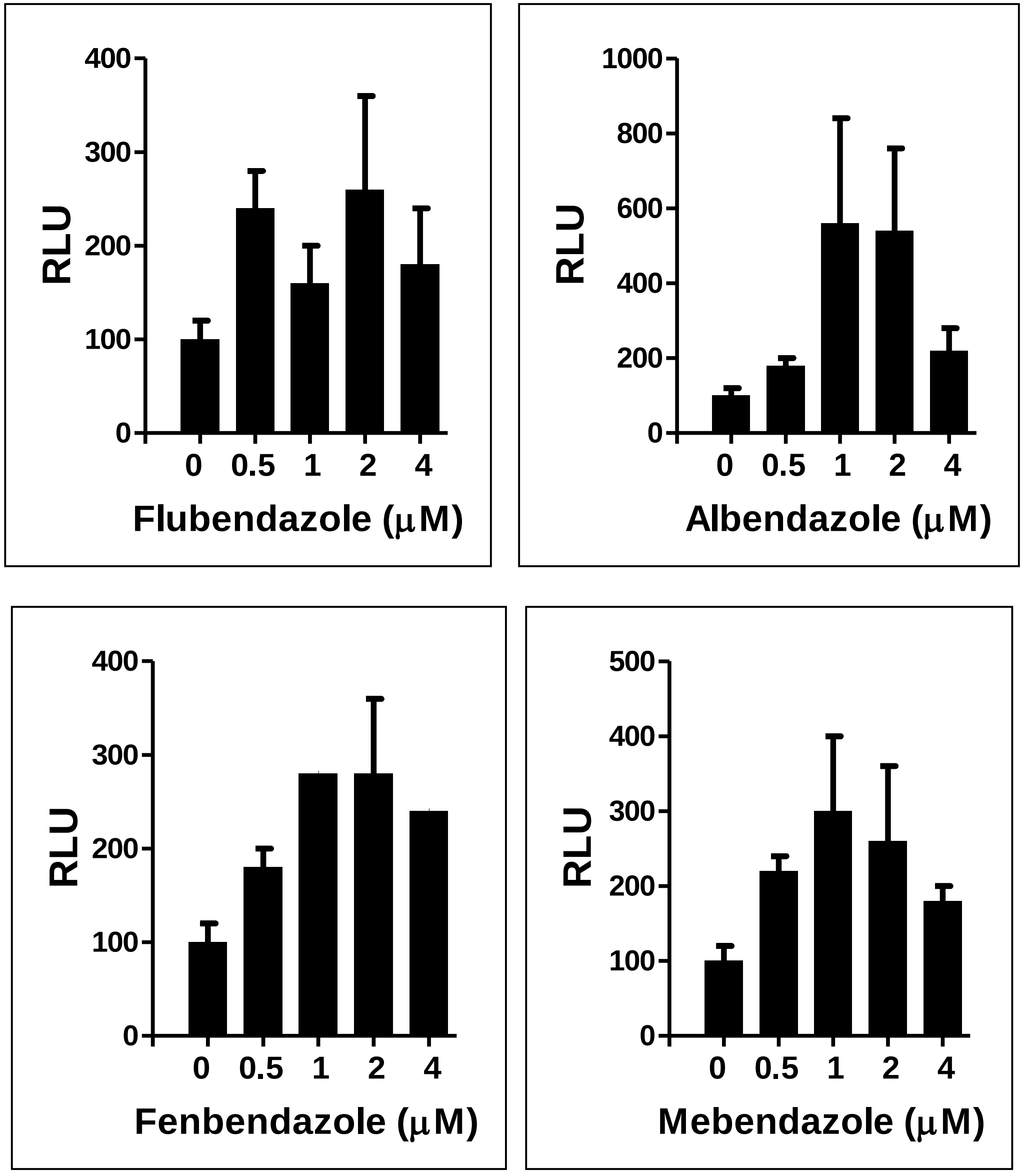

p53-Luc Agonists

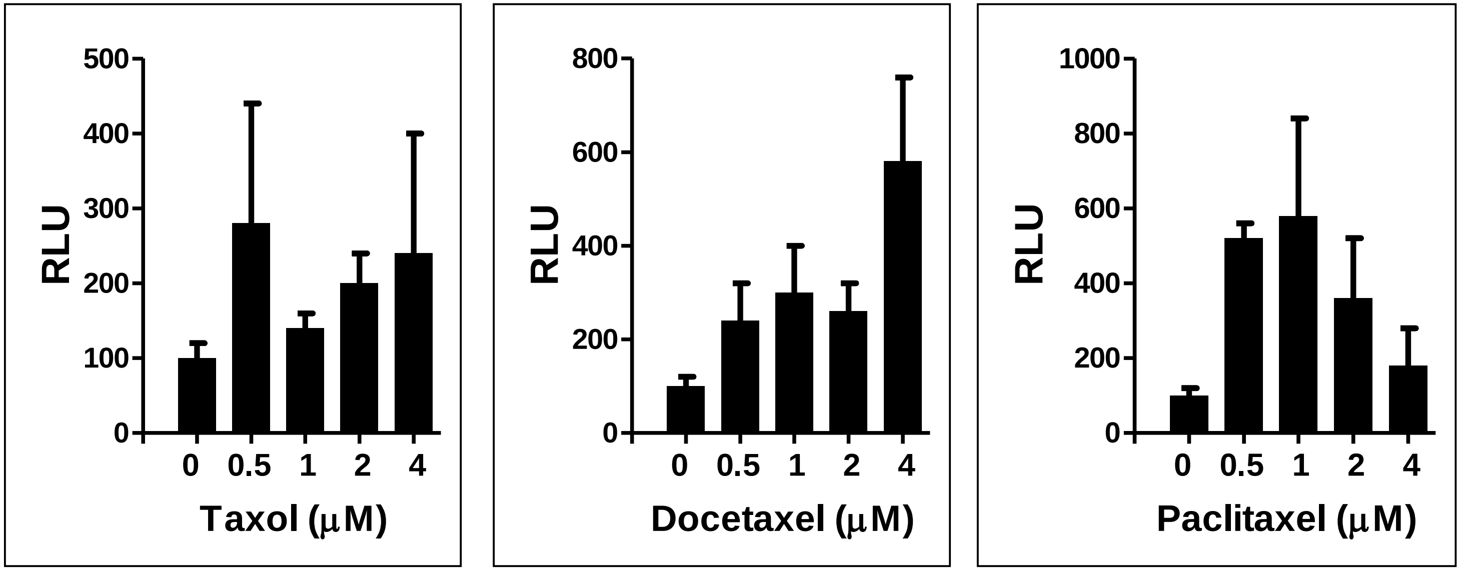

p53-Luc Agonists

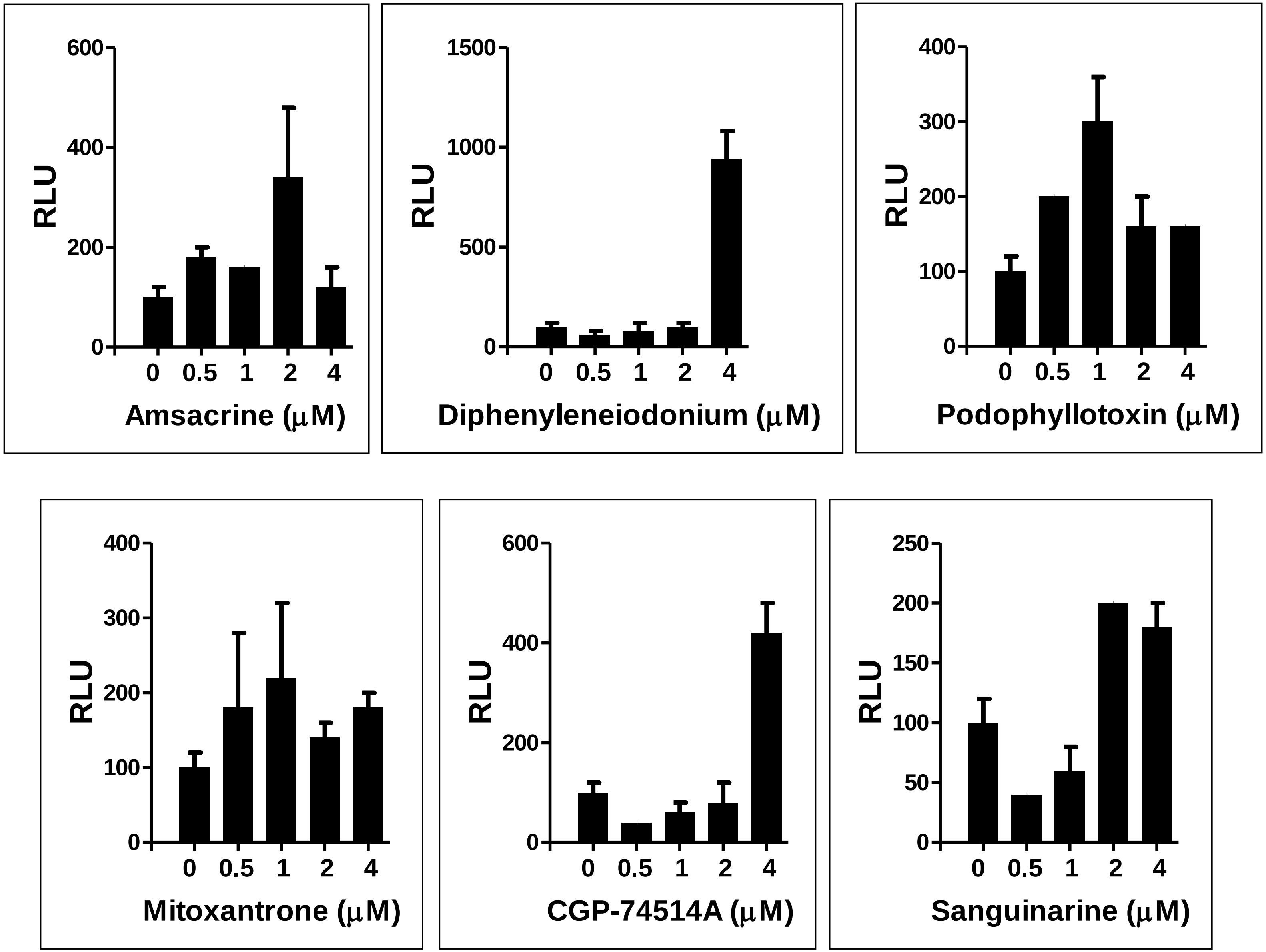

p53-Luc Synergists

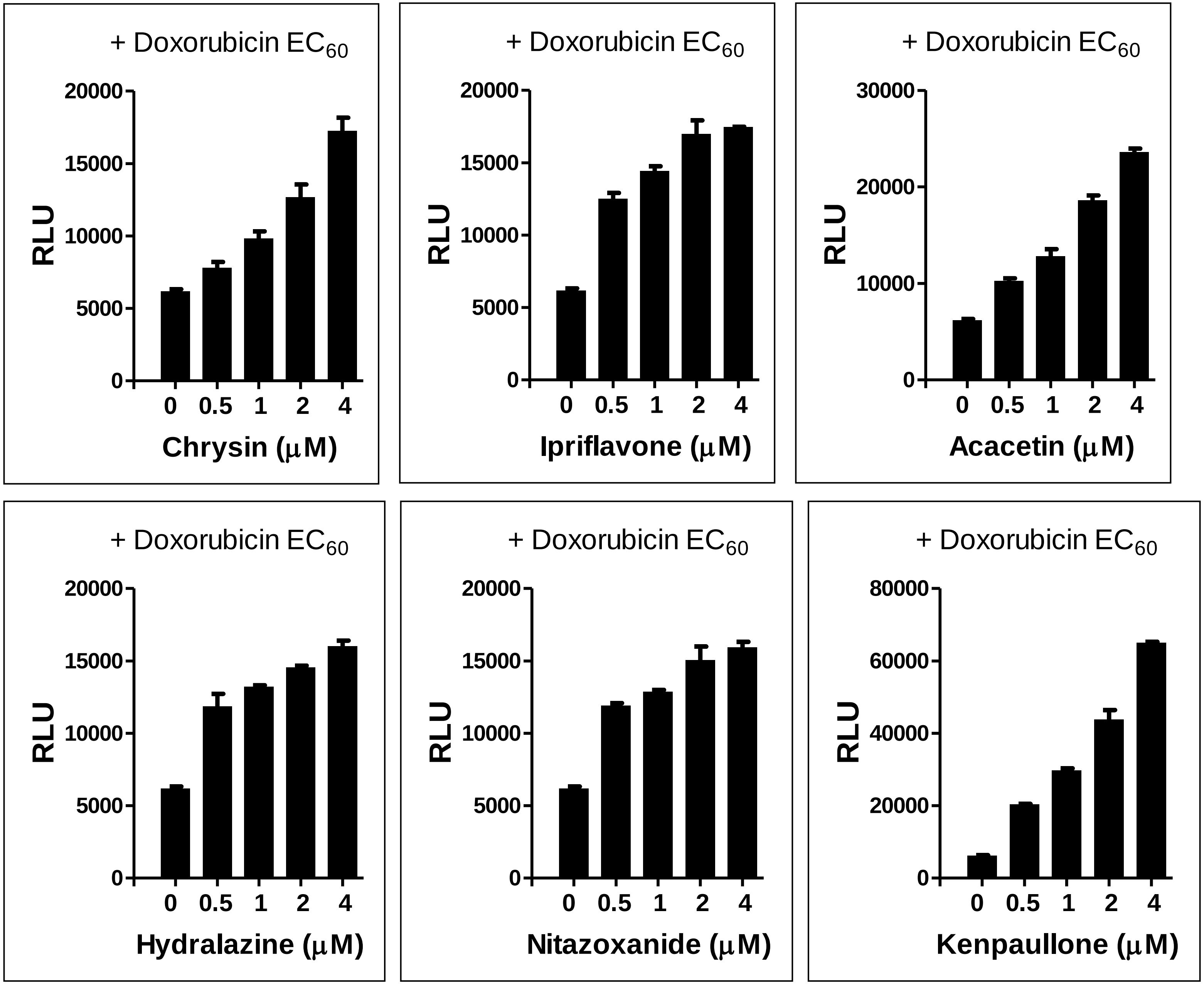

p53-Luc Synergists

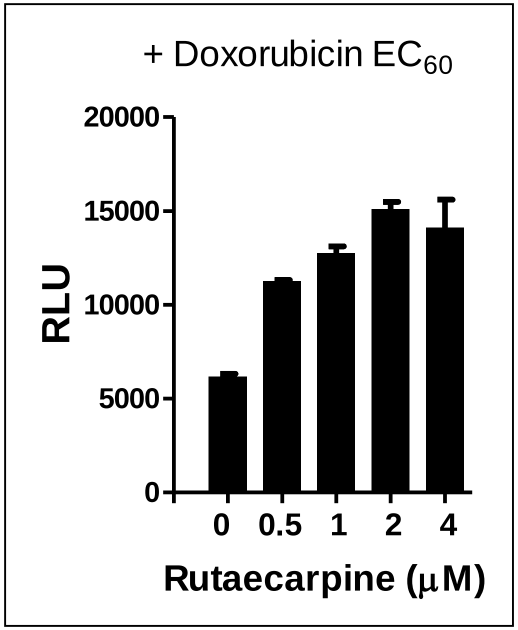

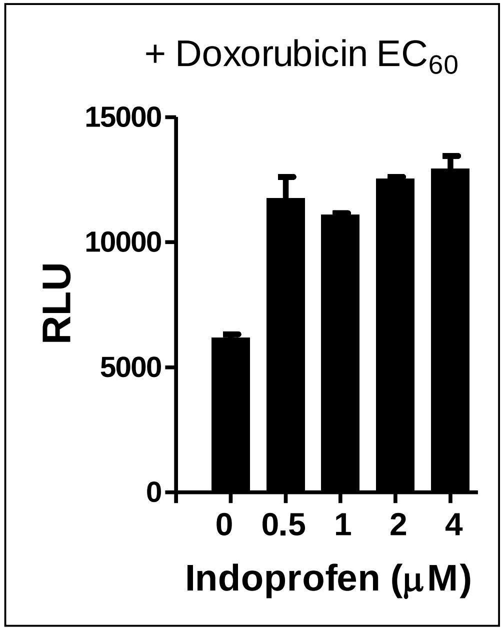

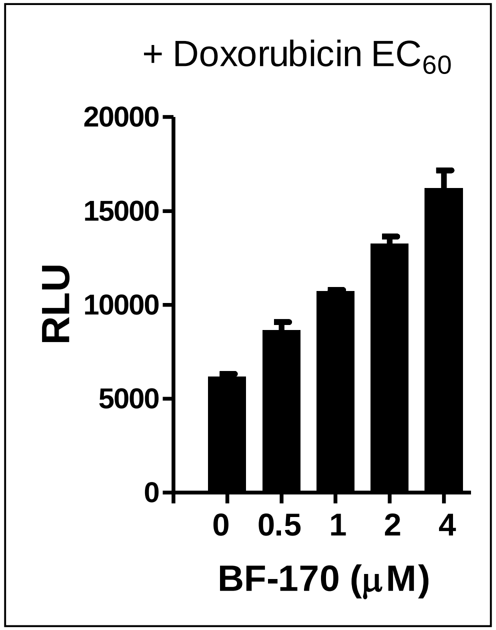

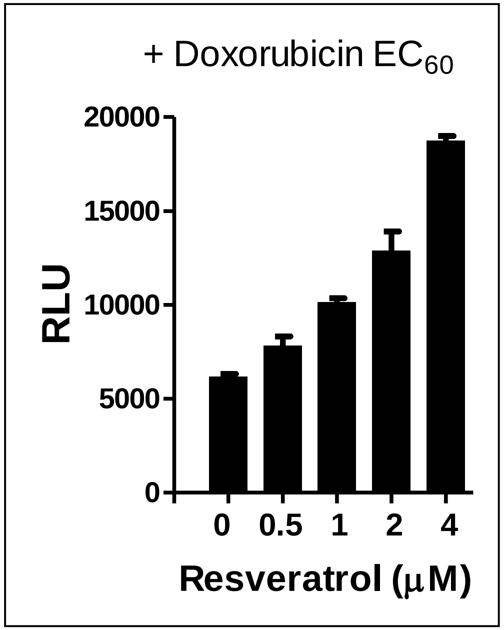

p53-Luc Agonists and Synergists

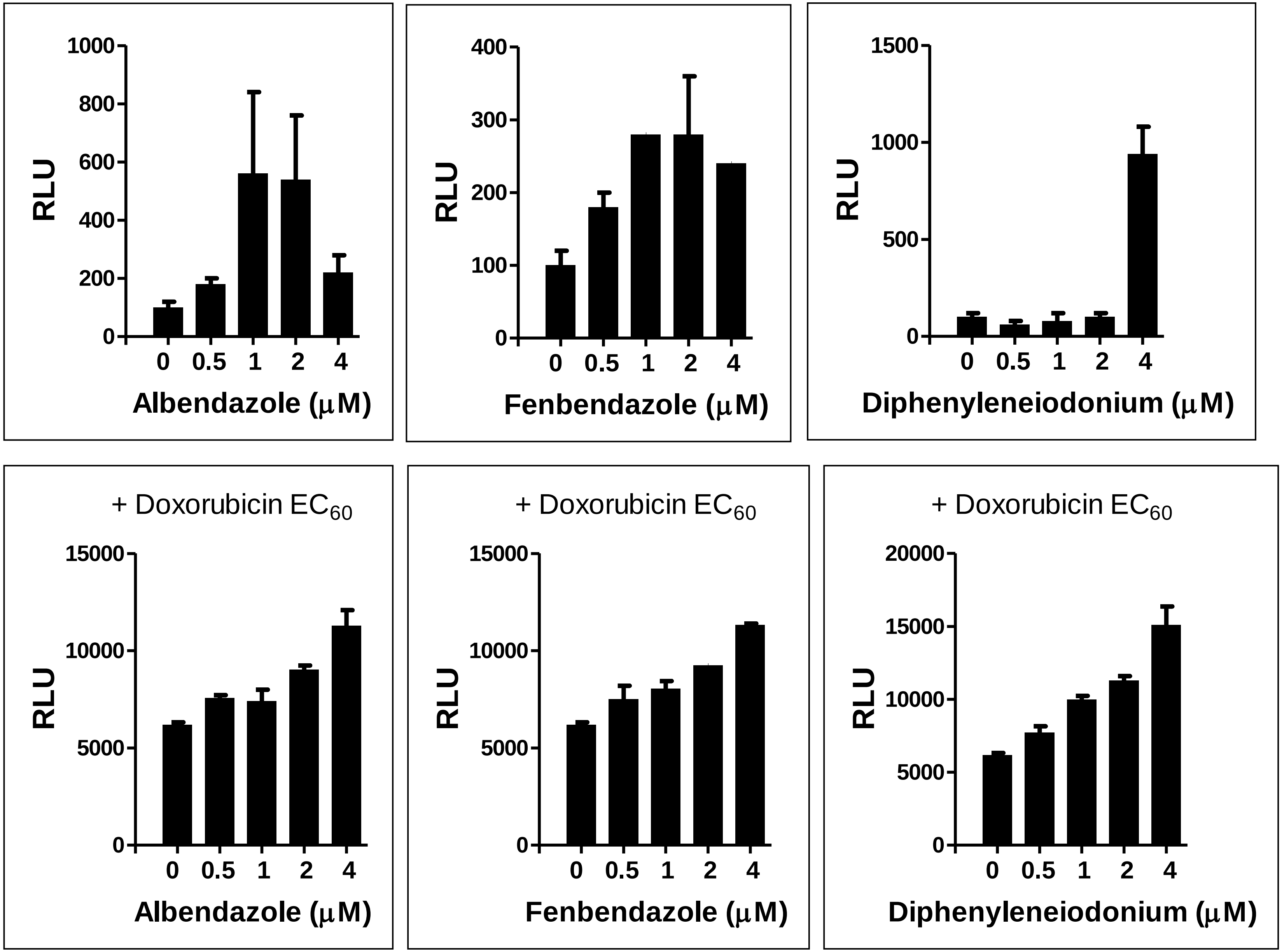

p53-Luc Antagonists

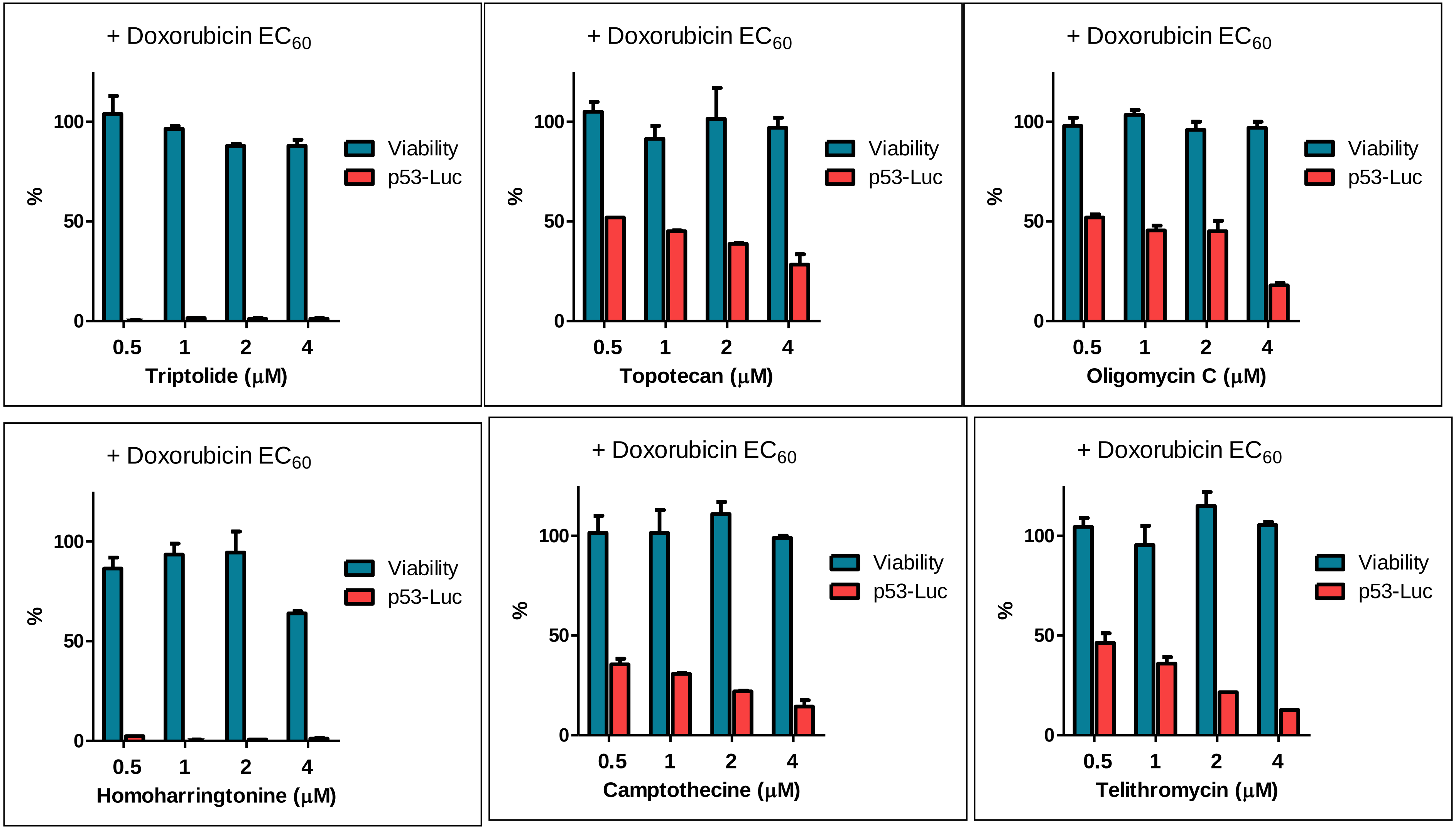

p53-Luc Antagonists

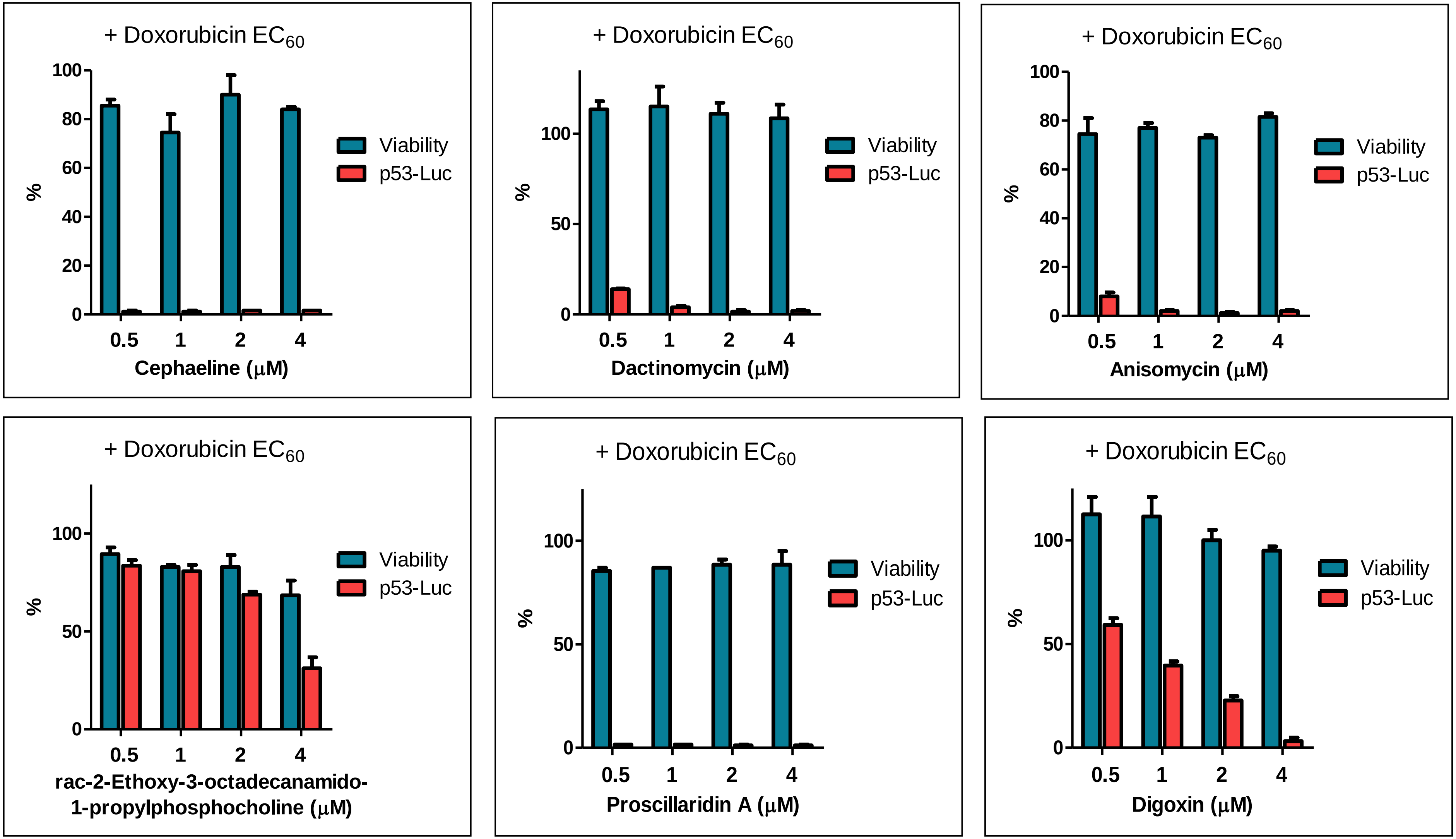

p53-Luc Antagonists

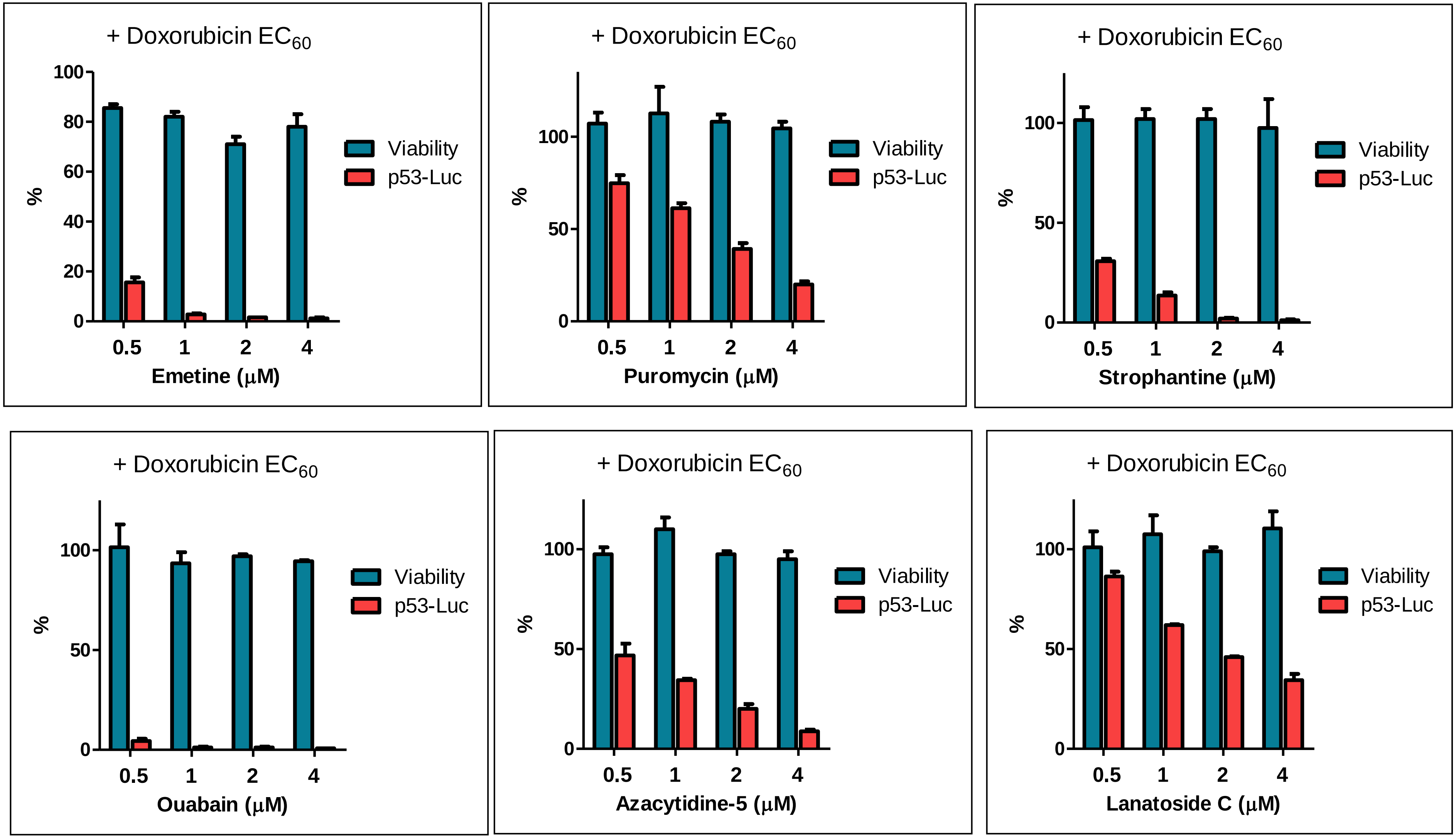

p53-Luc Antagonists

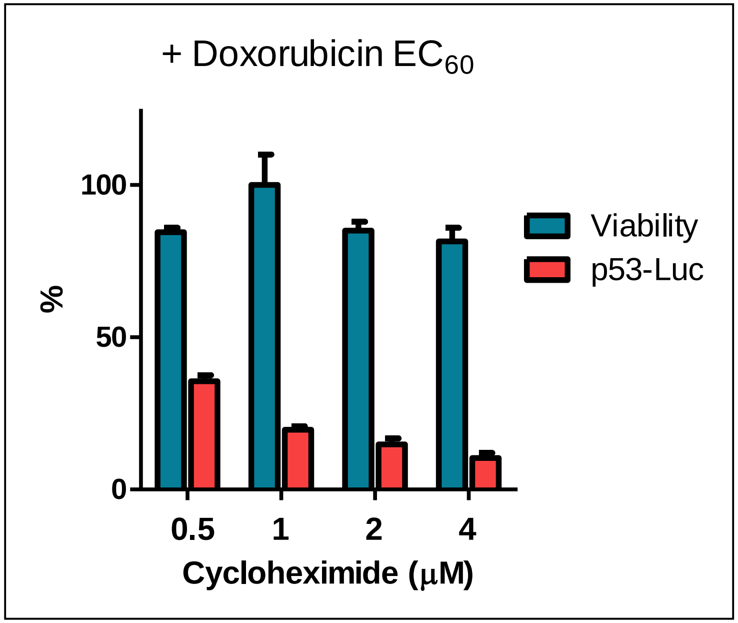

Supplement: Supplementary file 1 [file molecules-24-02152-s001.zip › SlaninovaFigureS1.pdf]
